# Methods for environmental change; an exploratory study

**DOI:** 10.1186/1471-2458-12-1037

**Published:** 2012-11-28

**Authors:** Gerjo Kok, Nell H Gottlieb, Robert Panne, Chris Smerecnik

**Affiliations:** 1 Work & Social Psychology, Maastricht University, Maastricht, the Netherlands; 2 Kinesiology & Health Education, University of Texas at Austin, Texas, USA; 3 Applied Psychology, Fontys Hogeschool, Eindhoven, the Netherlands

**Keywords:** Behavior change method, Environment, Health promotion, Intervention

## Abstract

**Background:**

While the interest of health promotion researchers in change methods directed at the target population has a long tradition, interest in change methods directed at the environment is still developing. In this survey, the focus is on methods for environmental change; especially about how these are composed of methods for individual change (‘Bundling’) and how within one environmental level, organizations, methods differ when directed at the management (‘At’) or applied by the management (‘From’).

**Methods:**

The first part of this online survey dealt with examining the ‘bundling’ of individual level methods to methods at the environmental level. The question asked was to what extent the use of an environmental level method would involve the use of certain individual level methods. In the second part of the survey the question was whether there are differences between applying methods directed ‘at’ an organization (for instance, by a health promoter) versus ‘from’ within an organization itself. All of the 20 respondents are experts in the field of health promotion.

**Results:**

Methods at the individual level are frequently bundled together as part of a method at a higher ecological level. A number of individual level methods are popular as part of most of the environmental level methods, while others are not chosen very often. Interventions directed at environmental agents often have a strong focus on the motivational part of behavior change.

There are different approaches targeting a level or being targeted from a level. The health promoter will use combinations of motivation and facilitation. The manager will use individual level change methods focusing on self-efficacy and skills. Respondents think that any method may be used under the right circumstances, although few endorsed coercive methods.

**Conclusions:**

Taxonomies of theoretical change methods for environmental change should include combinations of individual level methods that may be bundled and separate suggestions for methods targeting a level or being targeted from a level. Future research needs to cover more methods to rate and to be rated. Qualitative data may explain some of the surprising outcomes, such as the lack of large differences and the avoidance of coercion. Taxonomies should include the theoretical parameters that limit the effectiveness of the method.

## Background

In planning health promotion interventions, theories from the behavioral sciences are applied to, first, understand behavior and, second, change behavior. Such interventions apply theoretical methods for change, directed at the target population or at the environment. While the interest of health promotion researchers in methods directed at the target population has a long tradition, interest in methods directed at the environment is still developing. In the survey among health promotion experts reported here, the focus is on methods for environmental change; especially about how methods for environmental change may be composed of methods for individual change and how within one environmental level, organizations, methods may differ when applied by a health promoter and directed at the management or applied by the management directed at the employees.

### Theoretical methods for change

A theoretical method, or behavior change technique, is a general technique or process for influencing changes in the determinants of behavior of the target population or of behavior of the environmental decision maker [1,2] (see Table 1 for examples). Practical applications are specific techniques for practical use of theoretical methods in ways that fit the intervention population and the context in which the intervention will be conducted [1]. For example, a change objective for an intervention might be to increase adolescents’ self-efficacy to resist social pressure to use drugs. For the change objective of increasing self-efficacy, theoretical methods might include Modeling, Guided Practice with Feedback, and Reinforcement. One application for Modeling could be a videotaped step-by-step demonstration by adolescents of how to resist peer pressure in situations they commonly encounter. However, there may be environmental conditions relevant in this example. An environmental condition of adolescent drug use could be the availability of drugs for sale in neighborhoods where adolescents live, with an objective that the mayor would get police to actively enforce laws against neighborhood drug dealers. A change objective for this might be to increase the mayor’s positive outcome expectations, for example, that this enforcement will save children’s lives, be popular with constituents, be positively received by powerful groups in the city, and increase tourism to the city. The primary environmental theoretical method could be Advocacy, which might include methods of Information, Persuasion, Negotiation, and Coercion. One practical application of Advocacy might be for influential neighborhood activists to hold a breakfast meeting with the mayor, neighborhood constituents, and key city opinion leaders. The activists might present detailed case histories of neighborhood teens, along with pictures of open drug dealing on the street. If the mayor does not respond to this application, the group might undertake, as an additional method, Media Advocacy with an exposé story calling for action by the mayor on the local television channel as the application.

**Table 1:** **Methods and definitions (selected examples derived from Bartholomew, et al., 2011, chapter 6**[1]**)**

**Method**	**Definition**
*Methods at the Environmental Level (Bundling)*	
*Participatory Problem Solving [3]	Diagnosing the problem, generating potential solutions, developing priorities, making an action plan, and obtaining feedback after implementing the plan.
*Advocacy and Lobbying [4]	Arguing and mobilizing resources on behalf of a particular change; giving aid to a cause; active support for a cause or position.
Mobilizing Social Networks [5]	Encouraging social networks to provide informational, emotional, appraisal, and instrumental support.
*Organizational Diagnosis and Feedback [6]	Assessing of organizational structures and employees’ beliefs and attitudes, desired outcomes and readiness to take action, using surveys and other methods.
Community Development [7]	A form of community organization, based on consensus, in which power is shared equally and members engage together in participatory problem solving.
*Social Action [7]	A form of community organization, based in conflict, in which disenfranchised people wrest power from the official power.
*Forming Coalitions [8]	Forming an alliance among individuals or organizations, during which they cooperate in joint action to reach a goal in their own self-interest.
Agenda Setting [9]	Process of moving an issue to the political agenda for action; may make use of advocacy and media when initiated from outside government.
*Methods at the Individual Level (Bundling)*	
*Persuasive Communication [10]	Guiding individuals and environmental agents toward the adoption of an idea, attitude, or action by using arguments or other means.
*Modeling [11]	Providing an appropriate model being reinforced for the desired action.
*Feedback [12]	Giving information to individuals and environmental agents regarding the extent to which they are accomplishing learning or performance, or the extent to which performance is having an impact.
*Reinforcement/Punishment [12]	Providing reinforcement: linking a behavior to any consequence that increases the behavior’s rate, frequency or probability.Providing punishment: linking a behavior to any consequence that decreases the behavior’s rate, frequency or probability.
*Consciousness Raising [13]	Providing information, feedback, or confrontation about the causes, consequences, and alternatives for a problem or a problem behavior.
*Goal Setting [14]	Prompting planning what the person will do, including a definition of goal-directed behaviors that result in the target behavior.
*Facilitation [15]	Creating an environment that makes the action easier or reduces barriers to action.
*Information About Others’ Approval [16]	Providing information about what others think about the person’s behavior and whether others will approve or disapprove of any proposed behavior change.
*Resistance to Social Pressure [17]	Stimulating building skills for resistance to social pressure.
Guided Practice [11]	Prompting individuals to rehearse and repeat the behavior various times, discuss the experience, and provide feedback.
Individual Level and Environmental Level Methods (At and From)*	
Tailoring [18]	Matching the intervention or components to previously measured characteristics of the participant.
Direct Experience [19]	Encouraging a process whereby knowledge is created through the interpretation of experience.
Systems Change (Env.) [20]	Interacting with the environment to change the elements and relationship among elements of a system at any level, especially through dialogue with stakeholders, action, and learning through feedback.
Coercion (Env.) [21]	Attempting to control others against their will.
Technical Assistance (Env.) [22]	Providing technical means to achieve desired behavior.
Sense-Making (Env.) [23]	Leaders reinterpret and relabel processes in organization, create meaning through dialogue, and model and redirect change.
Team Building & Human Relations Training (Env.) [6]	Grouping development activities based on the values of human potential, participation, and development.
Structural Redesign (Env.) [24]	Change organizational elements such as formal statements of organizational philosophy, communication flow, reward systems, job descriptions, and lines of authority.
Increasing Stakeholder Influence (Env.) [25]	Increase stakeholder power, legitimacy, and urgency, often by forming coalitions and using community development and social action to change an organization’s policies.
Reporting, Social Planning [26]	Using information based on research to address issues.
Media Advocacy (Env.) [27]	Expose environmental agents’ behaviors in the mass media to order to get them to improve health related conditions. A type of advocacy.

*: Method is used in both parts of the study; definitions are only given once.

Table 1 provides the methods that were used in this study and their definitions. These definitions are originally based on theoretical descriptions in the literature, as indicated for each method. Preliminary definitions were formulated and send out to 50 colleagues in the field of health promotion and health psychology for evaluation and improvement. About 40 colleagues responded with suggestions and the authors of the 3rd edition of Bartholomew et al. [1] formulated the final definitions.

### Parameters for methods

**Figure 1:**
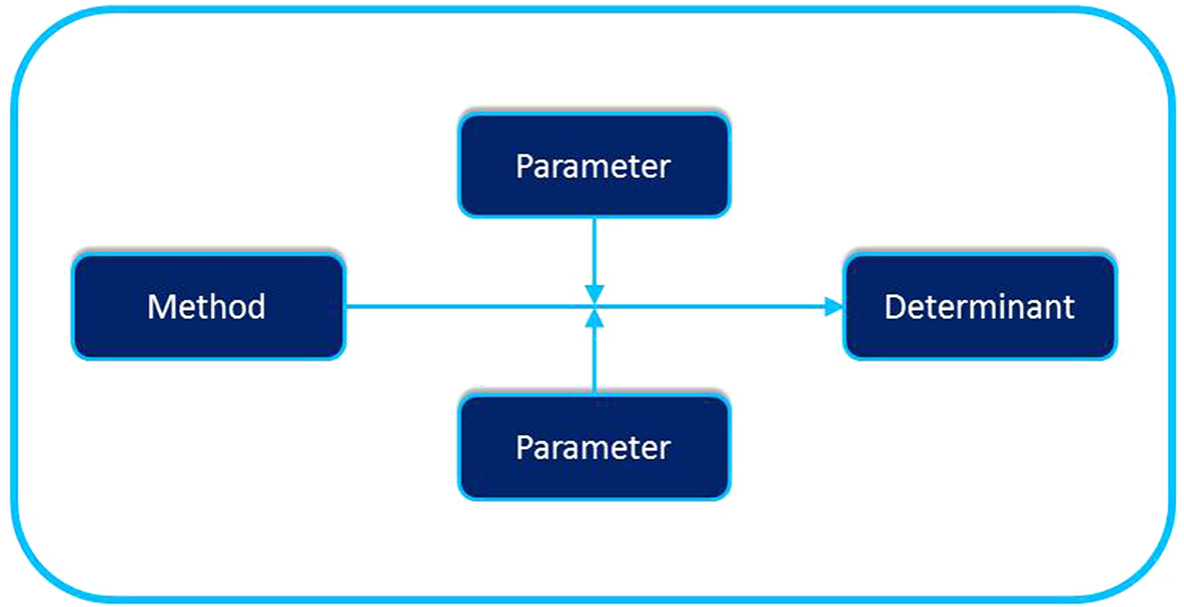
Parameters for Methods.

**Figure 2:**
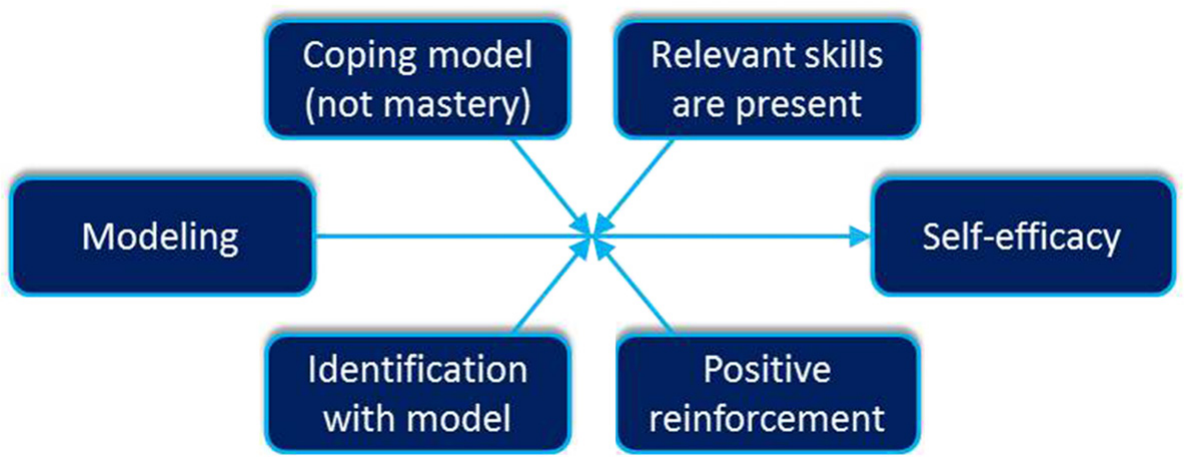
Parameters for Modeling.

There is an increasing interest in systematic descriptions or taxonomies of health promotion interventions, the theoretical methods they contain, and the determinants that are targeted for change [28]. However, most of these taxonomies focus on individual behavior change and only a few also include behavior change of environmental agents [1,29]. Moreover, translating methods into applications demands a sufficient understanding of the theory behind the method, especially the theoretical parameters under which the theoretical process is effective or not [30], see Figure 1. For example, Modeling is a strong method but only when certain parameters are met, for instance reinforcement of the modeled behavior [11], see Figure 2. People or environmental decision makers do not just behave in the desired manner because a model shows that behavior; they behave comparable to the model only when the model is reinforced for that particular behavior and when they expect to be reinforced in a similar way. Translating the method Modeling to a practical application includes taking care that in the actual program, from the perspective of the program participants, the model is reinforced. All theoretical methods have these parameters, which have to be taken into account when translating a method into a practical application.

### Ecological approach

Environmental conditions are not likely to be under the direct control of the individuals at risk for the health problem. They are controlled by decision makers, external agents such as peers, teachers, managers, and other gatekeepers. In the example above, the environmental agent was the mayor. In addition, environmental conditions may be at various environmental levels: interpersonal, organizational, community, and societal levels [31]. To select methods for environmental conditions, the first thing to do is to find out who may be in a position to make the expected change. The program planner has to identify the desired behaviors for the agent who will actually change the environmental condition. The health promoter then applies methods for influencing the determinants of the agent’s behavior using methods which are appropriate for changing determinants at environmental levels. For example (see Table 1), a basic method for all environmental levels is Advocacy, a method for the interpersonal level is Enhancing Network Linkages, for the organizational level Sense-Making, for the community level Social Action, and for the societal level Agenda Setting. Again, there are theoretical parameters for effectiveness of these environmental level methods, e.g. Agenda Setting requires appropriate timing; Social Action needs to start where the community is [1].

### Methods at environmental levels

Methods at the individual level can be directed toward agents at higher ecological levels. The theoretical process behind the method is the same; however, often the application of the method is somewhat different, depending on the target. For instance, in a study about interventions to change environmental conditions [31] persuasion was applied at various levels and originating from various levels. For example, in a project to decrease carbon dioxide transmission, the health promoter reported a Persuasive Communication approach that illustrated to businesses, corporations and other companies the advantages of approaching and dealing with the issue of carbon dioxide emissions. The health promoter showed them how carbon dioxide reduction is profitable and made it clear to companies that being environmentally friendly is positive for the company image. The potential effect on the image of and profit for the company are typical organizational level arguments.

### Bundling of methods

**Figure 3:**
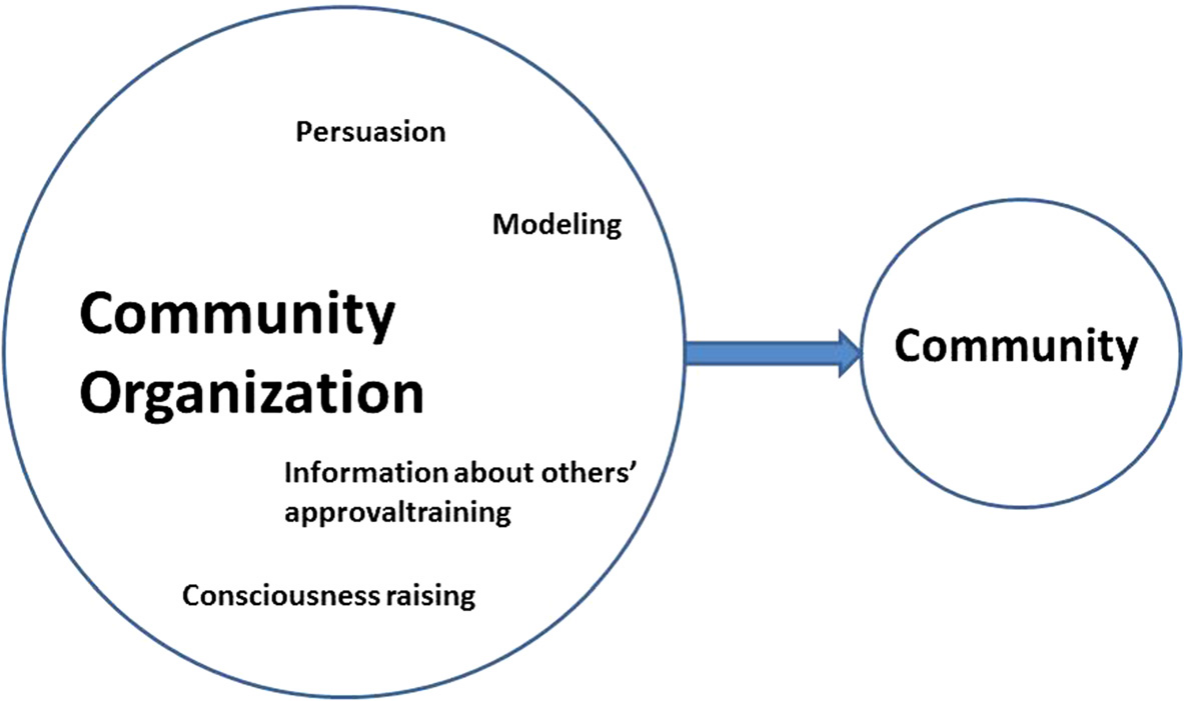
Bundling of individual methods in Community Development.

The focus of this exploratory study is on methods for environmental change; especially about how methods for environmental change may be composed of methods for individual change and how within one environmental level, organizations, methods may differ when applied by a health promoter and directed at the management or applied by the management directed at the employees. Methods at the individual level seem to be frequently bundled together as part of a change method at a higher ecological level. This is because environmental agents and organization and community members are also individuals and the determinants of their behaviors are similar to determinants of behavior at the individual level. The change target and the overall method, however, are specific to the environmental change level. For example Community Development can include the individual methods of Persuasion, Modeling, Consciousness raising, and Information about others’ approval; however, these methods are bundled together to accomplish a change in a community level problem and to increase community capacity, see Figure 3. Organizational Development, in fact, has been defined as the transfer of behavioral science knowledge to increase organizational effectiveness and the process resembles Behavioral Self-Regulation applied to the organizational level for example [32].

### Methods “At” and “From”

**Figure 4:**
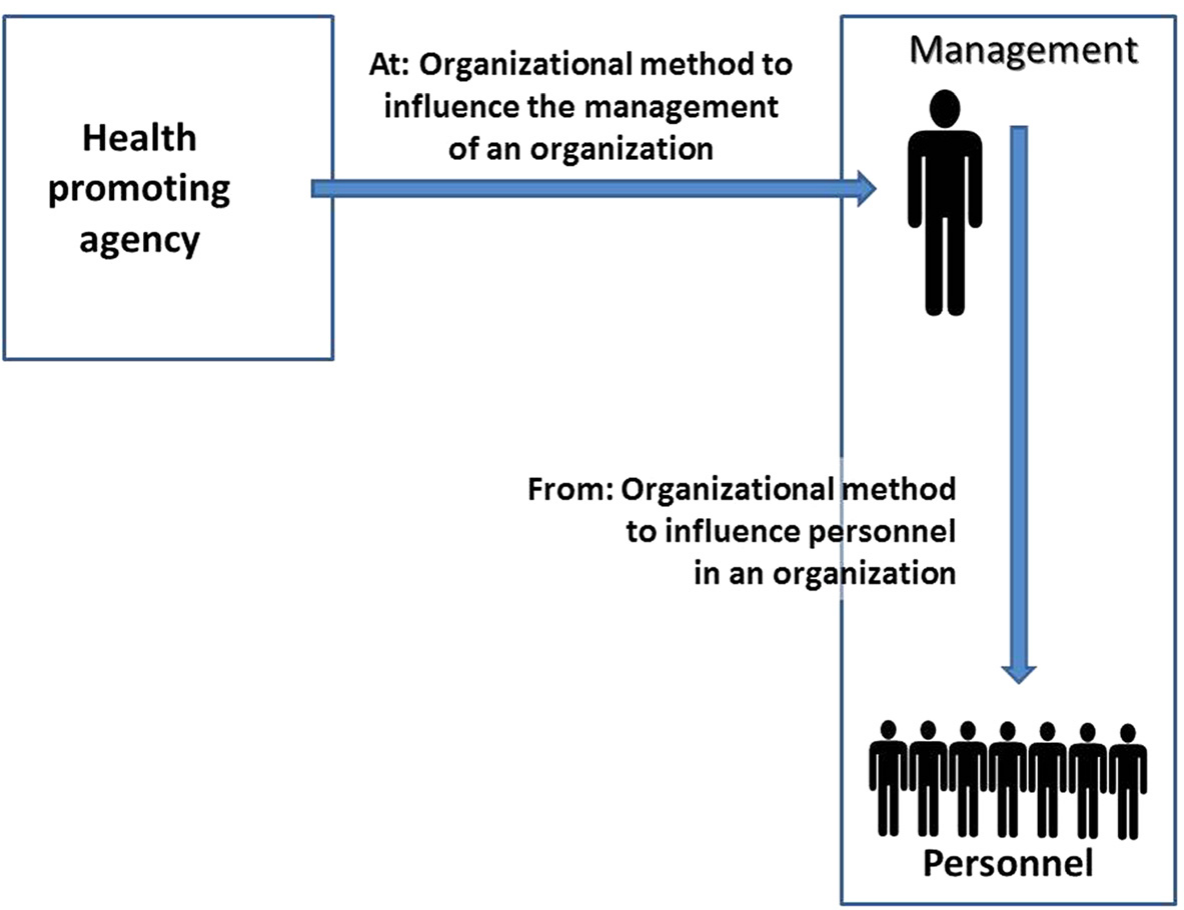
Methods ‘At’ and ‘From”.

Moreover, there may be different approaches targeting a level or being targeted from a level, see Figure 4. On the one hand, organizations may apply methods for improving the health of their employees, for instance, to increase physical activity [33]. Those methods might include Tailoring, Goal Setting, and Modeling. The activities are initiated by the management and are directed at the employees. On the other hand, health promoters and health-promoting organizations may apply methods to get organizations to start health-promoting activities as in the earlier examples above, for instance reducing carbon dioxide transmissions. A national voluntary heart organization may try to encourage companies to facilitate physical activity programs for their employees. Methods that are used include Persuasive Communication, Advocacy and Lobbying, Organizational Modeling, and Facilitation. These activities are initiated outside the organization, usually by a health promoter, and are directed at the organization, often the management. An interesting parallel to this process can be found in the research tradition of corporate social responsibility [34]. An example of corporate social responsibility is a community focusing on a company with respect to environmental pollution. In this case, the community initiates an activity using the method of Coalition Formation. These communities may have themselves been the focus of health-promoting organizations applying the method of Community Organizing.

### Hypotheses

The two-part survey reported here constitutes a study among health promotion experts on “Bundling” and “At and From”. The first part concerns the “bundling” of methods, addressing which of the methods at the individual level are most popular when applying environmental-level methods. The second part focuses on the organizational level and deals with the differences between methods used when a desired organization change originates outside of the focal organization (methods from a health promoter directed “at” the organization) or inside the organization (methods “from” the management directed at the employees). The exploratory research questions are:

1. Are methods at the individual level frequently bundled together as part of a change method at a higher ecological level? If so, which methods are bundled for the various environmental methods?

2. Are there different approaches targeting a level or being targeted from a level? If so, what are those differences?

## Methods

### Procedure and measures

This online expert survey was conducted using LimeSurvey [35]. There were two parts. The first part dealt with examining the possibility of bundling individual level methods to methods at the environmental level. The question asked was always to what extent the use of an environmental level method would involve the use of certain individual level methods, for example: “In your opinion, to what extent would using the method "Social Action" involve the use of the following methods at the individual level?”. For each of eight environmental methods presented, the participants (all experts in health promotion) could choose between five answering options for each of ten individual methods. The individual level methods were the same for all eight environmental level methods (see Table 1). The five options ranged from “This method would definitely not be used” (equal to 1) via “This method may or may not be used” (equal to 3) to “This method would definitely be used” (equal to 5). In the second part of the survey the question was whether there are differences between applying methods when change methods are directed at an organization (for instance, a health promoter targeting an organization) versus from within an organization itself (from the management). Methods were rated on two questions, the first dealing with “at”: “How useful would this method be if a health promoter tries to get the management of an organization to adopt and implement health promotion activities with their employees?”, and the second question dealing with “from”: “How useful would this method be if the management of an organization tries to promote a healthy lifestyle in their employees?”. There were 25 methods to be rated and both methods at the individual and at the environmental level were used (see Table 1). The answering options were similar, however this time the term was “useful” instead of “used”. For all methods in both parts of the survey definitions were provided [1] (see Table 1) which could easily be obtained by hovering the mouse cursor over a method. Methods were chosen based on a) theoretical clarity, b) high consensus about the definition of the method, c) diversity over determinants, e.g.: attitudes, social influences and self-efficacy, and diversity over environmental levels: interpersonal, organizational, community and policy levels, and on d) preliminary expectations of the research team that the theory behind the method already suggests the expected bundling or the specific use for At or From. The study was approved by the Ethics Committee of the School of Psychology and Neuroscience, Maastricht University.

### Respondents

All of the respondents are experts in the field of health promotion, and were contacted through the social network of the first two authors, mostly from the USA, Europe, Canada, and Australia. Respondents were involved in health promotion research, had published about the use of theoretical methods in interventions and/or about the role of the environment in health promotion, and had experience with developing health promotion programs in real life. The survey was distributed by sending e-mails containing a link to the survey. From the 50 experts invited, 20 provided complete data.

### Analyses

Means and standard deviation were computed, and T-tests were performed when comparisons were made between means.

## Results

### Bundling

For the environmental level method ‘Participatory Problem Solving’, the individual level methods Goal Setting, Facilitation, and Feedback are rated fairly high (4.0-4.5), while Reinforcement or Punishment, Guided Practice, and Resistance to Social Pressure are rated fairly low (2.5-3.0), see Table 2. This means that the experts agree that the individual-level methods Goal Setting, Facilitation and Feedback are very likely to be used when ‘Participatory Problem Solving’ is applied. On the other hand, Reinforcement or Punishment, Guided Practice and Resistance to Social Pressure are found to be not as popular when using ‘Participatory Problem Solving’.

**Table 2:** **Participatory Problem Solving and Advocacy and Lobbying, means and standard deviations (bold:≥ 4.0; *Italics: ≤ 3.0*)**

***Participatory Problem Solving***			***Advocacy and Lobbying***		
**Method**	**M**	**SD**	**Method**	**M**	**SD**
**Goal Setting**	**4.50**	**0.83**	**Persuasive Communication**	**4.55**	**0.69**
**Facilitation**	**4.35**	**0.88**	**Info About Others' Approval**	**4.50**	**0.83**
**Feedback**	**4.30**	**0.80**	**Consciousness Raising**	**4.30**	**0.73**
**Consciousness Raising**	**4.10**	**1.07**	Facilitation	3.70	0.98
Info About Others' Approval	3.60	0.99	Modeling	3.60	0.94
Persuasive Communication	3.55	1.50	Feedback	3.55	0.83
Modeling	3.20	1.32	Goal Setting	3.55	1.00
*Resistance to Social Pressure*	*3.00*	0.97	Resistance to Social Pressure	3.45	1.05
*Guided Practice*	*2.95*	1.28	Reinforcement/Punishment	3.25	0.79
*Reinforcement/Punishment*	*2.65*	*1.18*	*Guided Practice*	*2.60*	*1.10*

For ‘Advocacy and Lobbying’, the individual level methods Persuasive Communication, Information About Other’s Approval and Consciousness Raising are most popular with the experts (all around 4.5), while Guided Practice seems to be of little importance (just above 2.5), see Table 2.

‘Mobilizing Social Networks’ has fairly evenly distributed ratings: Information About Other’s Approval, Facilitation and Persuasive Communication are ranked highest at above 4.0. Guided Practice is again ranked lowest at 3.0, see Table 3.

**Table 3:** **Mobilizing Social Networks and Organizational Diagnosis and Feedback, means and standard deviations (bold:≥ 4.0; *Italics: ≤ 3.0*)**

***Mobilizing Social Networks***			***Organizational Diagnosis and Feedback***		
**Method**	**M**	**SD**	**Method**	**M**	**SD**
**Info About Others' Approval**	**4.20**	**0.70**	**Feedback**	**4.55**	**0.89**
**Facilitation**	**4.15**	**1.09**	**Consciousness Raising**	**4.00**	**1.17**
**Persuasive Communication**	**4.10**	**0.85**	Facilitation	3.65	1.35
Consciousness Raising	3.95	0.89	Goal Setting	3.30	1.26
Goal Setting	3.80	1.11	Info About Others' Approval	3.30	1.38
Modeling	3.75	0.91	*Persuasive Communication*	*2.80*	*1.20*
Feedback	3.70	0.98	*Reinforcement/Punishment*	*2.65*	*1.09*
Resistance to Social Pressure	3.60	0.94	*Modeling*	*2.60*	*1.14*
Reinforcement/Punishment	3.25	0.85	*Guided Practice*	*2.50*	*1.05*
*Guided Practice*	*3.00*	*1.12*	*Resistance to Social Pressure*	*2.40*	*0.94*

Not surprisingly, for ‘Organizational Diagnosis and Feedback’, Feedback is rated very highly at 4.55, followed by Consciousness Raising with 4.00. Resistance to Social Pressure and Guided Practice are ranked lowest at 2.4 and 2.5, respectively, see Table 3.

For the environmental level method ‘Community Development’, Consciousness Raising, Facilitation, Goal Setting, and Information About Other’s Approval are all likely to be used (4.0-4.5). Guided Practice is rated just below and Reinforcement or Punishment just above 3.0, which means that these methods may or may not be used, see Table 4.

**Table 4:** **Community Development and Social Action, means and standard deviations (bold:≥ 4.0; *Italics: ≤ 3.0*)**

***Community Development***			***Social Action***		
**Method**	**M**	**SD**	**Method**	**M**	**SD**
**Consciousness Raising**	**4.40**	**0.68**	**Consciousness Raising**	**4.55**	**0.69**
**Facilitation**	**4.35**	**1.09**	**Persuasive Communication**	**4.50**	**0.69**
**Goal Setting**	**4.15**	**1.04**	**Info About Others' Approval**	**4.15**	**0.99**
**Info About Others' Approval**	**4.10**	**0.79**	**Modeling**	**4.00**	**0.73**
Persuasive Communication	3.90	1.07	Resistance to Social Pressure	3.90	1.12
Feedback	3.85	1.09	Facilitation	3.85	1.27
Modeling	3.70	1.08	Reinforcement/Punishment	3.70	1.17
Resistance to Social Pressure	3.25	1.12	Goal Setting	3.70	1.13
Reinforcement/Punishment	3.15	1.23	Feedback	3.65	1.09
*Guided Practice*	*2.95*	*1.23*	*Guided Practice*	*2.70*	*1.22*

For ‘Social Action’, Consciousness Raising and Persuasive Communication are most popular at just above, respectively, exactly 4.5. Again, Guided Practice is the lowest at 2.7 and therefore not as likely to be applied, see Table 4.

When ‘Forming Coalitions’ is the chosen environmental method, Persuasive Communication is rated the highest at 4.3, followed by Consciousness Raising, Goal Setting, Facilitation and Information About Other’s Approval, which are all just above 4.0; Guided Practice is rated as rather unlikely at 2.7, see Table 5.

**Table 5:** **Forming Coalitions and Agenda Setting, means and standard deviations (bold:≥ 4.0; *Italics: ≤ 3.0*).**

***Forming Coalitions***			***Agenda Setting***		
**Method**	**M**	**SD**	**Method**	**M**	**SD**
**Persuasive Communication**	**4.30**	**0.73**	**Persuasive Communication**	**4.40**	**0.88**
**Consciousness Raising**	**4.15**	**0.75**	**Consciousness Raising**	**4.10**	**1.02**
**Goal Setting**	**4.15**	**0.88**	Goal Setting	3.90	1.02
**Facilitation**	**4.10**	**0.79**	Info About Others' Approval	3.90	1.21
**Info About Others' Approval**	**4.10**	**1.07**	Facilitation	3.80	1.24
Feedback	3.95	1.05	Modeling	3.55	1.05
Modeling	3.75	0.91	Feedback	3.40	1.10
Reinforcement/Punishment	3.35	1.23	Reinforcement/Punishment	3.40	1.19
*Resistance to Social Pressure*	*3.00*	*1.08*	Resistance to Social Pressure	3.10	1.21
*Guided Practice*	*2.70*	*1.03*	*Guided Practice*	*2.65*	*1.23*

The last method at the environmental level is ‘Agenda Setting’. For this method, Persuasive Communication is ranked highest (4.4) followed by Consciousness Raising (4.1) while Guided Practice is, again, the method considered least appropriate (2.65), see Table 5.

The average popularity of individual-level methods likely to be used over all methods at the environmental level is presented in Table 6. Consciousness Raising is most often regarded as useful (4.19). Persuasive Communication is second (4.01), followed by Facilitation (3.99), Information About Other’s Approval (3.98), Goal Setting (3.88), Feedback (3.87), Modeling (3.52), Resistance to Social Pressure (3.21), and Reinforcement or Punishment (3.18). Guided Practice comes in last at 2.76, which is still close to the “May or may not be used” mark of 3.0.

**Table 6:** **Overall average popularity, means and standard deviations (bold:≥ 4.0; *Italics: ≤ 3.0*)**

***Overall Average Popularity***					
**Method**	**M**	**SD**	**Method**	**M**	**SD**
**Consciousness Raising**	**4.19**	**0.89**	Feedback	3.87	1.04
**Persuasive Communication**	**4.01**	**1.12**	Modeling	3.52	1.08
Facilitation	3.99	1.14	Resistance to Social Pressure	3.21	1.12
Info About Others' Approval	3.98	0.98	Reinforcement/Punishment	3,18	1.11
Goal Setting	3.88	1.12	*Guided Practice*	*2,76*	*1.15*

### At and From

In the “At and From” part of the survey, multiple evaluations were given by the experts regarding the use of different methods, see Table 7. The top three useful methods when (a health promoter is) targeting an organization (“At”) are Persuasive Communication (4.85), Consciousness Raising (4.6), and Facilitation (4.5). When an organization’s employees are targeted from within (“From” its management), the most popular methods are Feedback (4.65), Goal Setting and Modeling (each at 4.55). The method ranked lowest by the experts and, thus, the least useful one is Coercion (At: 1.95, From: 2.1), followed by Social Action (At: 2.9, From: 2.6).

**Table 7:** **Popularity of methods “At” and “From”, means and standard deviations (bold:≥ 4.0; *Italics: ≤ 3.0*)**

***“At”***			***“From”***		
**Method**	**M**	**SD**	**Method**	**M**	**SD**
**Persuasive Communication**	**4.85**	**0.37**	**Feedback**	**4.65**	**0.59**
**Consciousness Raising**	**4.60**	**0.82**	**Modeling**	**4.55**	**0.60**
**Facilitation**	**4.50**	**0.89**	**Goal Setting**	**4.55**	**0.69**
**Technical Assistance**	**4.45**	**0.76**	**Facilitation**	**4.45**	**1.00**
**Organ. Diagnosis and Feedback**	**4.45**	**0.83**	**Tailoring**	**4.40**	**0.99**
**Feedback**	**4.35**	**0.88**	**Technical Assistance**	**4.35**	**0.81**
**Info About Others' Approval**	**4.35**	**0.93**	**Persuasive Communication**	**4.15**	**1.14**
**Modeling**	**4.30**	**0.66**	**Consciousness Raising**	**4.15**	**1.18**
**Participatory Problem Solving**	**4.25**	**0.72**	**Direct Experience**	**4.10**	**1.07**
**Advocacy and Lobbying**	**4.25**	**0.72**	**Reinforcement/Punishment**	**4.05**	**0.76**
**Tailoring**	**4.20**	**1.01**	**Info About Others' Approval**	**4.05**	**0.89**
**Goal Setting**	**4.15**	**0.99**	Systems Change	3.95	1.15
Systems Change	3.95	1.15	Participatory Problem Solving	3.90	0.91
Direct Experience	3.70	1.13	Structural Redesign	3.70	1.13
Team Building and HR Training	3.70	1.03	Sense-Making	3.65	1.31
Increasing Stakeholder Influence	3.70	1.13	Team Building and HR Training	3.65	0.88
Reporting, Social Planning	3.70	1.22	Resistance to Social Pressure	3.60	0.88
Reinforcement/Punishment	3.65	1.04	Organ. Diagnosis and Feedback	3.45	1.19
Forming Coalitions	3.65	0.93	Reporting, Social Planning	3.20	1.11
Sense-Making	3.55	1.00	Increasing Stakeholder Infl.	3.10	1.02
Structural Redesign	3.45	1.15	Forming Coalitions	3.10	1.37
Media Advocacy	3.35	1.18	*Media Advocacy*	*2.95*	*1.36*
Resistance to Social Pressure	3.30	1.13	*Advocacy and Lobbying*	*2.85*	*1.31*
*Social Action*	*2.90*	*1.33*	*Social Action*	*2.60*	*1.27*
*Coercion*	*1.95*	*1.05*	*Coercion*	*2.10*	*1.21*

Methods that are much more likely to be used when targeting an organization from the outside compared to from within are Advocacy and Lobbying (At: 4.25 versus From: 2.85; t = 4.63, p < .001), Organizational Diagnosis and Feedback (At: 4.45 versus From: 3.45; t = 3.25, p < .001), Persuasive Communication (At: 4.85 versus From: 4.15; t = 2.77, p < .01) and Forming Coalitions (At: 3.65 versus From 3.10; t = 2.77, p < .01). A method more likely to be used when an organization is targeted from the inside is Direct Experience (At: 3.7 versus From: 4.1; t = −2.18, p << .04), see Table 8.

**Table 8:** Popularity of methods “At” and “From”, means and standard deviations, t-tests and p-values

	***“At”***		***“From”***			
**Method**	**M**	**SD**	**M**	**SD**	**t**	**P**
Persuasive Communication	4.85	0.37	4.15	1.14	2.77	.01
Tailoring	4.20	1.01	4.40	0.99	−0.66	.52
Modeling	4.30	0.66	4.55	0.69	−1.56	.14
Feedback	4.35	0.93	4.65	0.59	−1.19	.25
Reinforcement/Punishment	3.65	1.04	4.05	0.89	−2.03	.06
Facilitation	4.50	0.89	4.45	1.00	0.25	.80
Consciousness Raising	4.60	0.82	4.15	1.18	1.69	.11
Direct Experience	3.70	1.22	4.10	1.07	−2.18	.04
Info about others’ approval	4.35	0.88	4.05	0.76	1.19	.25
Resistance to Social Pressure	3.30	1.13	3.60	0.88	−0.88	.39
Goal Setting	4.15	0.99	4.55	0.60	−1.71	.10
Systems Change	3.95	1.15	3.95	1.15	0.00	.00
Participatory Problem Solving	4.25	0.72	3.90	0.91	1.44	.17
Coercion	1.95	1.05	2.10	1.21	−0.57	.58
Advocacy and Lobbying	4.25	0.72	2.85	1.31	4.63	.00
Technical Assistance	4.45	0.83	4.35	0.81	0.57	.58
Sense-making	3.55	1.00	3.65	0.88	−0.44	.67
Organ. Diagnosis and Feedback	4.45	0.76	3.45	1.19	3.25	.00
Team Building	3.70	1.13	3.65	1.31	0.16	.87
Structural Redesign	3.45	1.15	3.70	1.13	−0.69	.50
Increasing Stakeholder Influence	3.70	1.13	3.10	1.37	1.71	.10
Social Action	2.90	1.33	2.60	1.27	1.45	.16
Forming Coalitions	3.65	0.93	3.10	1.02	2.77	.01
Reporting, Social Planning	3.70	1.03	3.20	1.11	1.88	.08
Media Advocacy	3.35	1.18	2.95	1.36	1.57	.13

## Discussion

The survey reported here constituted a study among health promotion experts with two parts, one on Bundling and one on ‘At’ and ‘From’. The exploratory research questions of this study were

1. Are methods at the individual level frequently bundled together as part of a change method at a higher ecological level? If so, which methods are bundled for the various environmental methods?

2. Are there different approaches targeting a level or being targeted from a level? If so, what are those differences?

### Bundling

Methods at the individual level are frequently bundled together as part of a change method at a higher ecological level. For example ‘Participatory Problem Solving’ is often comprised of Goal Setting, Facilitation, Feedback, and Consciousness Raising, while ‘Social Action’ is often comprised of Consciousness Raising, Persuasive Communication, Information About Others’ Approval, and Modeling. Moreover, a number of individual level methods are popular as part of most of the environmental level methods, while some others are obviously not chosen very often. Consciousness Raising and Persuasive Communication are the two most popular methods to be bundled, followed by Facilitation. This suggests that interventions directed at the environmental agents often have a strong focus on the motivational part of behavior change, awareness and attitude change; in combination with helping agents to realize the desired behavior by Facilitation. Goal Setting, Feedback, and especially Modeling are very popular in interventions targeting individual level change (see also Table 7), but do not seem to be applied that often in environmental level interventions. Guided Practice is the least popular method chosen at the environmental level, which is understandable as it is mostly used in face-to-face counseling within a setting where the target person has already a positive intention to change. Reinforcement/Punishment as a method is also not popular and some participants explained that they would never use punishment and therefore avoided this combination of methods.

What does it mean that methods at the environmental level seem to be composed of individual level methods? The major difference between environmental-level and individual-level methods is the target: environmental agent versus individual. At the individual level, often methods are also combined in a program. Of course, the content is different, for example politicians react to potential election success, managers react to profit, and newspaper editors react to news value, while at the individual level, personal losses and gains are most relevant. However, the bundling of methods at the environmental level has a special character, the setting or collectivity: social networks, organizations, communities, and political networks. Nevertheless, we need more insight in what makes environmental-level methods more than just combinations of individual-level methods.

### ‘At’ and ‘From’

There are different approaches targeting a level or being targeted from a level, in this study for the organizational level. For example, a health promoter targeting (the management of) an organization will probably use Persuasive Communication, Consciousness Raising, and Organizational Diagnosis and Feedback, in combination with Facilitation and Technical Assistance. Again, we see at this level a focus on a combination of motivation (awareness and attitude change) with practical support (making the desired behavior easier to do). On the other hand, a manager of an organization targeting employees’ behavior change will probably use Feedback, Modeling, Goal Setting and Tailoring, also in combination with Facilitation and Technical Assistance. This situation seems to be more comparable to individual level change: the focus is less on awareness and attitude change, but more on self-efficacy and skills. Typical organizational level methods for change are also used but less often, for example Systems Change, Participatory Problem Solving, Structural Redesign, Sense-Making, and Team Building & Human Relations Training.

Only a few methods show substantial differences in use, for example Advocacy and Lobbying, Organizational Diagnosis and Feedback, Persuasive Communication, and Forming Coalitions are clearly seen as more popular with the health promoter targeting an organization than with a manager targeting employees. For Direct Experience, it is the other way around. Obviously, most respondents think that any method may be used under the right circumstances as almost all methods score above the mean of the scale. It is also interesting to note that health promoters targeting an organization will not often use Coercion, Social Action, Media Advocacy, or Forming Coalitions. In reality, health promoters would probably use those methods; however, they would use them not directly but indirectly through relevant stakeholders [25]: Coercion through government, Social Action through communities, Media Advocacy through the media, and Forming Coalitions with other (health promotion) organizations.

What does it mean that with managers the focus is on motivation, while with employees the focus is on action? One explanation is that there are different goals: deciding about others versus own behavior change. It would be interesting to study situations where the behavior change of the employees is relatively easy and motivational change is enough, in combination with situations in which managers’ decisions are quite difficult to perform and they need extensive skills.

### Relevance

The outcomes of this study are also relevant for implementation of health promotion programs. Very often interventions are implemented by professionals in organizations such as hospitals and schools, by, for example, nurses and teachers. Health promotion organizations develop programs directed at patients or students, using individual level methods, e.g. Goal Setting and Guided Practice. They also develop training programs for nurses and teachers combining individual level methods with environmental level methods, e.g., Participatory Problem Solving and Team Building. Finally they have to convince the school and hospital management to adopt the program and assure that nurses and teachers have enough time and skills, by applying environmental methods such as Sense-Making and Structural Redesign.

### Limitations

The number of respondents is quite low and there is minimal information about the experts’ specific expertise and experiences. The inclusion criteria were broad and data were collected from experts with various backgrounds such as health psychology, epidemiology, and community development. Not all experts may have been familiar with each of the theoretical methods we presented to them in the survey, even though definitions were made easily available. There may be differences between on the one hand what the experts think and on the other hand real life practice. Also for practical reasons, the number of theoretical methods in the two parts of the survey was limited, which makes the results informative but not exhaustive. Some of the methods and definitions were inadequate, for example the combination of reinforcement/punishment. This study was focused on the organizational level, and future studies should also focus on other levels: interpersonal, community, and policy level. Nevertheless, despite these limitations, the basic questions of this study about bundling and about targeting or being targeted can be answered affirmatively.

## Conclusions

Methods at the individual level are indeed frequently bundled together as part of a change method at a higher ecological level, and there are indeed different approaches targeting a level or being targeted from a level. Taxonomies of theoretical methods for environmental change should include combinations of individual level methods that may be bundled. And also separate suggestions for methods targeting a level or being targeted from a level. Future research needs to cover more methods to rate and more methods to be rated, while exploring alternative ways to allocate individual to environmental level methods. Also, more qualitative data may explain some of the surprising outcomes of this study, such as the lack of large differences and the avoidance of coercion and punishment while in other environmental change traditions these methods are suggested to be effective [25].

Finally, taxonomies of environmental change methods should include the theoretical parameters that limit the effectiveness of the theoretical process; in this case a combination of parameters associated with the various individual level methods bundled in the environmental level method. For example, Participatory Problem Solving is defined as: diagnosing the problem, generating potential solutions, developing priorities, making an action plan, and obtaining feedback after implementing the plan (see Table 1). Bartholomew et al. [1] mention the following parameters: requires willingness by the health promoter or convener to accept the participants as equals and as having a high level of influence; requires target group to possess appropriate motivation and skills (p. 347). In Table 2, the four most often used individual level methods are: Goal Setting, Facilitation, Feedback, and Consciousness Raising. These four methods have their own parameters which are different from those for participatory decision making. For example, the parameters for Goal Setting are: Commitment to the goal; goals that are difficult but available within the individual’s skill level (p. 344). The parameters for Consciousness Raising are: can use feedback and confrontation; however, raising awareness must be quickly followed by increase in problem-solving ability and (collective) self-efficacy (p. 333). Program planners, who apply environmental methods for change, need to be aware of the parameters under which that method may be effective. And, as a consequence of the bundling, they also need to be aware of the parameters for the individual level methods that are part of the environmental method. Other taxonomies that have been published [29] or are currently being developed, should not only focus on correct definitions but also on adequate parameters for use.

## Competing interests

The authors declare that they have no competing interests.

## Authors’ contributions

GK and NHG conceived of the study; GK, NHG and CS participated in the design of the study; RP carried out the study and performed the analyses; GK and RP drafted the manuscript; and all authors read the manuscript, provided comments and approved of the final manuscript.

## Pre-publication history

The pre-publication history for this paper can be accessed here:

http://www.biomedcentral.com/1471-2458/12/1037/prepub
